# Implementing radical cure diagnostics for malaria: user perspectives on G6PD testing in Bangladesh

**DOI:** 10.1186/s12936-021-03743-w

**Published:** 2021-05-12

**Authors:** Nora Engel, Cristian Ghergu, Mohammad Abdul Matin, Mohammad Golam Kibria, Kamala Thriemer, Ric N. Price, Xavier C. Ding, Rosalind E. Howes, Benedikt Ley, Sandra Incardona, Mohammad Shafiul Alam

**Affiliations:** 1 Department of Health, Ethics & Society, Research School for Public Health and Primary Care (CAPHRI), Faculty of Health, Medicine & Life Sciences, Maastricht University, Maastricht, Netherlands; 2 Infectious Diseases Division, International Centre for Diarrhoeal Disease Research, Bangladesh (icddr,b), Dhaka, Bangladesh; 3 Global and Tropical Health Division, Menzies School of Health Research, Charles Darwin University, Darwin, NT Australia; 4 Centre for Tropical Medicine and Global Health, Nuffield Department of Medicine, University of Oxford, Oxford, UK; 5 Mahidol-Oxford Tropical Medicine Research Unit, Faculty of Tropical Medicine, Mahidol University, Bangkok, Thailand; 6 Foundation for Innovative New Diagnostics (FIND), Geneva, Switzerland

**Keywords:** G6PD testing, Point of care diagnostics, Bangladesh, Users, *P. vivax*

## Abstract

**Background:**

The radical cure of *Plasmodium vivax* requires treatment with an 8-aminoquinoline drug, such as primaquine and tafenoquine, to eradicate liver hypnozoite stages, which can reactivate to cause relapsing infections. Safe treatment regimens require prior screening of patients for glucose-6-phosphate dehydrogenase (G6PD) deficiency to avoid potential life-threatening drug induced haemolysis. Testing is rarely available in malaria endemic countries, but will be needed to support routine use of radical cure. This study investigates end-user perspectives in Bangladesh on the introduction of a quantitative G6PD test (SD Biosensor STANDARD™ G6PD analyser) to support malaria elimination.

**Methods:**

The perspectives of users on the SD Biosensor test were analysed using semi-structured interviews and focus group discussions with health care providers and malaria programme officers in Bangladesh. Key emerging themes regarding the feasibility of introducing this test into routine practice, including perceived barriers, were analysed.

**Results:**

In total 63 participants were interviewed. Participants emphasized the life-saving potential of the biosensor, but raised concerns including the impact of limited staff time, high workload and some technical aspects of the device. Participants highlighted that there are both too few and too many *P. vivax* patients to implement G6PD testing owing to challenges of funding, workload and complex testing infrastructure. Implementing the biosensor would require flexibility and improvisation to deal with remote sites, overcoming a low index of suspicion and mutual interplay of declining patient numbers and reluctance to test. This approach would generate new forms of evidence to justify introduction in policy and carefully consider questions of deployment given declining patient numbers.

**Conclusions:**

The results of the study show that, in an elimination context, the importance of malaria needs to be maintained for both policy makers and the affected communities, in this case by ensuring *P. vivax*, PQ treatment, and G6PD deficiency remain visible. Availability of new technologies, such as the biosensor, will fuel ongoing debates about priorities for allocating resources that must be adapted to a constantly evolving target. Technical and logistical concerns regarding the biosensor should be addressed by future product designs, adequate training, strengthened supply chains, and careful planning of communication, advocacy and staff interactions at all health system levels.

**Supplementary Information:**

The online version contains supplementary material available at 10.1186/s12936-021-03743-w.

## Background

Twenty one countries across the Asia–Pacific region have set a target for eliminating malaria by 2030 [1]. Whereas *Plasmodium falciparum* case numbers have decreased significantly over the last decade, the success with *Plasmodium vivax* infections has been much lower [2, 3]. In contrast to *P. falciparum, P. vivax* forms dormant liver stages (hypnozoites) that can reactivate weeks to months after the initial infection causing recurrent febrile illness and cumulative risk of morbidity and mortality. In most endemic areas, more than 60% of vivax malaria is attributable to relapsing infections [4]. The 8-aminoquinolines primaquine (PQ) and tafenoquine (TQ) are the only available drugs capable of killing *P. vivax* hypnozoites. While well tolerated in most patients, both can cause severe and potentially fatal drug induced hemolysis in patients with glucose-6-phosphate dehydrogenase (G6PD) deficiency [5]. To improve its tolerability, PQ is administered over a prolonged 14 day course, but such a long treatment course is associated with poor adherence [6–8]. The World Health Organization (WHO) recommends that all patients should be tested for G6PD deficiency (G6PDd) prior to administration of PQ or TQ, and deficient patients offered alternative treatment regimens [9]. Shorter treatment regimens, including TQ or a 7-day high daily dose PQ regimens, are efficacious [10, 11], but increase the risk of drug-induced haemolysis and thus the need for prior screening for G6PDd. Increased availability of G6PD testing is, therefore, essential for safe administration of radical cure treatments that will be required for the timely elimination of *P. vivax*.

While the population at risk of malaria has declined over the last decade in Bangladesh, approximately 17 million population across 13 districts continue to be at risk of infection [12, 13], with highest numbers reported from the multi-ethnic Chittagong Hill Tracts Districts (CHT) in the Southeast of the country. In parallel to the decline in overall malaria cases in the country, the proportion of malaria episodes due to *P. vivax* has increased steadily, currently contributing approximately 20% of all cases [14–17]. PQ is provided for the radical cure of *P. vivax,* but patients are not tested for G6PDd prior to treatment. The prevalence of G6PDd is highly heterogeneous in Bangladesh [18], and its diagnosis is challenging [19]. The reference method is spectrophotometry but this requires a well-established laboratory infrastructure, which is rarely available in remote areas where the majority of malaria occurs [20, 21]. The G6PD gene is X-linked. Whilst hemizygous males or homozygous females are severely deficient (< 30% enzyme activity), heterozygous females can have intermediate enzyme activity (30–80%) [22, 23]. Qualitative point of care diagnostics are simple to use but categorize individuals above and below 30% enzyme activity and, therefore, do not identify females with intermediate deficiency who are also at risk of drug-induced haemolysis [24]. A quantitative assay is a much safer and more equitable tool to guide radical cure treatment decisions. A novel handheld biosensor (STANDARD™ G6PD, SD Biosensor, Republic of Korea) [25, 26], has been developed for use in point-of-care settings in endemic areas that could potentially address this shortfall.

The Bangladesh National Malaria Elimination Programme (NMEP) is currently considering novel approaches to G6PD diagnostics, supported by a dynamic research programme. If implemented, the biosensor would likely support the introduction of TQ or shorter PQ treatment courses. To assess the feasibility of implementing the biosensor in Bangladesh from the perspective of various users, a qualitative research study was undertaken to investigate user perspectives and practices of G6PD diagnostics [27, 28]. However, considerations around specific treatment courses and *P. vivax* case management as a whole were beyond the scope of the study, but have been the focus of another recent study [29]. This paper reports the different considerations and practices at clinic, health worker and policy levels that influence the introduction ofG6PD diagnostics for new radical cure therapeutics.

## Methods

### Study setting

The study focused on the healthcare level represented by Upazila Health Complexes (UHC) and District Hospitals (DH) in Bangladesh, which operate under the Ministry of Health. These settings were selected in consultation with NMEP for this study because if a quantitative G6PD test to support *P. vivax* radical cure is introduced to Bangladesh, it is likely to be at these levels of the health system, at least initially. UHCs provide primary health care with inpatient and outpatient services including laboratory diagnoses and operative treatment [30], with laboratories generally led by a trained laboratory technician (LT), named ‘medical technologist’ in Bangladesh. Other UHC staff include doctors, nurses and sub-assistant community medical officers (SACMO) who assist doctors, perform malaria rapid diagnostic tests (RDTs) and conduct patient consultations [31].

At the community level where most malaria patients seek care, non-governmental organization (NGO) and government community workers diagnose malaria using RDTs. At UHC and DHs, LTs use microscopy, but when unavailable, RDTs are used. Since 2008, the RDT used in Bangladesh is able to detect both *P. vivax* and *P. falciparum* [32]. The recommended treatment for *P. vivax* is chloroquine (CQ) for three days, and PQ at a total dose of 3.5 mg/kg administered over 14 days. This treatment regimen is provided at all levels of the health system without prior testing for G6PDd.

### Training workshops and focus group discussions

**Fig. 1:**
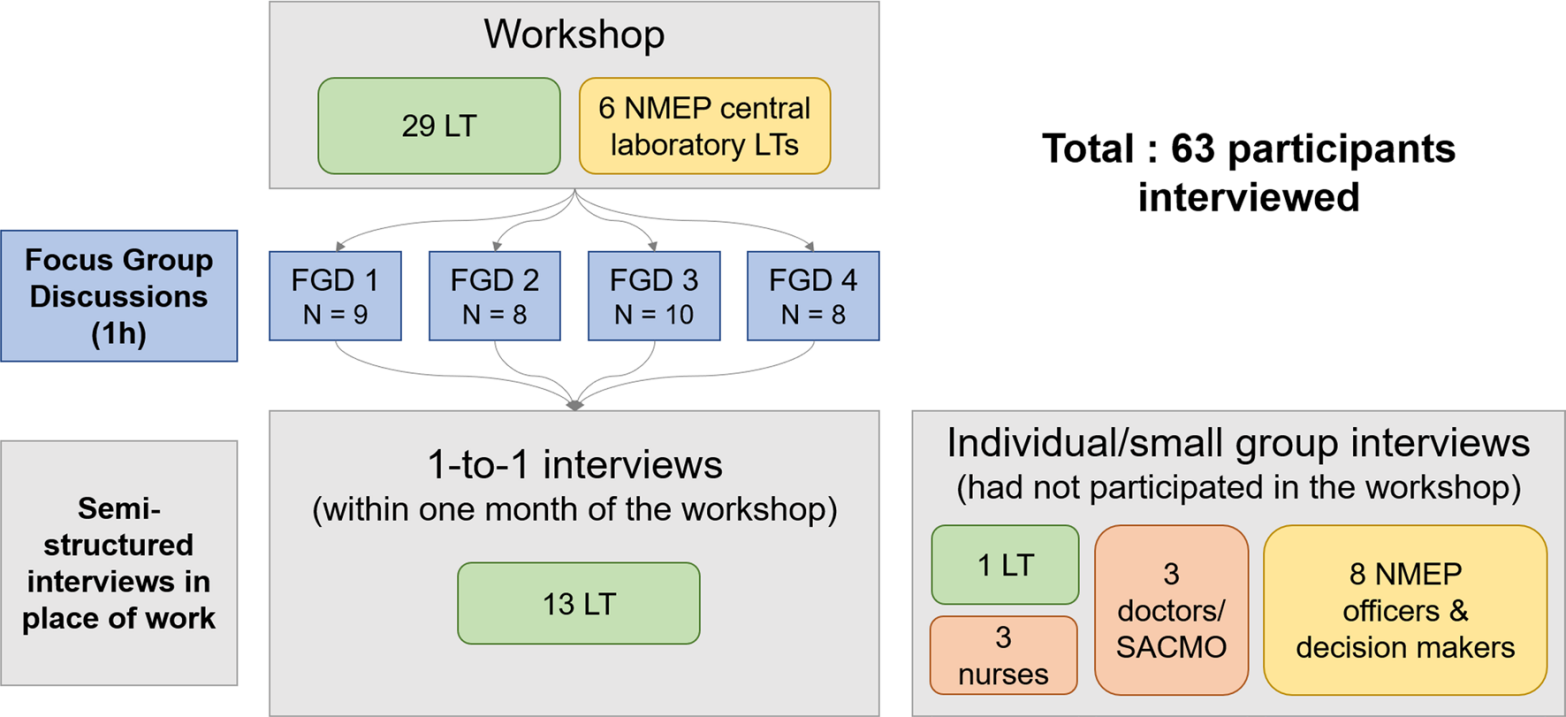
Summary of data collection activities: focus group discussions and interviews. *LT* laboratory technician, *FGD* focus group discussion, *SACMO* sub-assistant community medical officers

Data collection took place during October and November 2019 and included observation of training workshops, focus group discussions and semi-structured interviews (Fig. 1). In October 2019, two one-day training workshops were conducted by the International Centre for Diarrheal Disease Research (icddr,b), in collaboration with the Menzies School of Health Research (Menzies) and the Foundation for Innovative New Diagnostics (FIND), to train LTs on the use of the biosensor and then evaluate their proficiency. As per suggestion by the NMEP, the training focused on LTs who will likely be conducting the biosensor in the case of implementation. Workshop participants were given a presentation on G6PD, available G6PD diagnostics and a demonstration on how to use the biosensor. Participants then had the opportunity to practice operating the test in groups of 5–6 over a period of 90 min.

Four Focus Group Discussions (FGDs) were then conducted with groups of 8–10 workshop participants willing to participate (total 35) to understand their perceptions of introducing the biosensor into routine use in Bangladesh. The FGDs used semi-structured discussion guides (Additional file 1) and post-it note exercises to facilitate expression of opinions.

### Semi-structured interviews

To deepen insights and follow-up emerging themes from the four FGDs, 23 individual semi-structured interviews were conducted with a total of 28 participants (20 health workers, including 13 of the workshop participants, and 8 programme officers/ decision-makers). Three interviews involved multiple respondents (more specifically, two nurses, three LTs, and three programme officers were each interviewed together). Seven health care workers who had not participated in the training, including one LT, three doctors and three nurses who would need to order the test and act on its results, were interviewed to further explore topics raised during the FGDs, understand the participants’ practices and routines in providing care and to contextualize their perspectives on the diagnostic. These interviews were conducted in their place of work. The interview guides (Additional file 2) included topics such as diagnosing and treating malaria, PQ treatment and related risks, interactions with patients, changes in clinical practice, the training workshop (for LTs only), as well as preferences and challenges regarding the biosensor.

Finally, the programme officers and decision-makers were interviewed to understand the knowledge and perceptions towards G6PD testing at a policy level, as well as facilitating factors and barriers to implementing a new test and associated policy changes [with adjusted interview guides to reflect those aims (Additional file 3)].

### Study participants

This study focused on laboratory personnel and decision-makers, but also included clinicians and nurses. Workshop participants were selected by the NMEP. Other interviewed participants were purposively sampled to cover important steps in the diagnostic process. Owing to time and resource-constraints, they were limited to the public health system and approached based on convenience through personal contacts. Future research should include community level providers, NGOs as well as patients which were not included in this study.

The workshop participants included 29 LTs from sub-district UHCs and DHs across the malaria endemic area of Chittagong Division in the south-east of Bangladesh, along the Myanmar border, and six participants from the national malaria reference laboratory in Dhaka, the capital. Only two of the participants had used the biosensor previously.

The interviewed programme officers and decision-makers included previous and current officers acting in senior programme management positions, surveillance, monitoring and evaluation experts, and consultants of the malaria programme and other institutions (Table 1).

**Table 1:** Participant overview

	Professional role code	Interview participants	FGD1	FGD2	FGD3	FGD4	Total
Laboratory technician	LT	14 (12 interviews)	9	8	10	8	49
Nurse	Nurse	3 (2 interviews)					3
Doctor (+ SACMO)	Doctor	3 (3 interviews)					3
Programme officers and decision makers*	Programme officer	8 (6 interviews)					8
TOTAL		28 (23 interviews)					63

^*^Includes staff or consultants from the National Malaria Elimination Programme (NMEP), IEDCR (Institute of Epidemiology, Disease Control and Research), CDC (Communicable Disease Control), and BRAC (Building Resources across Communities)

*LT* laboratory technician, *FGD* Focus Group Discussion, *SACMO* sub-assistant community medical officers

### Data collection and analysis

All FGDs and interviews were conducted by two social scientists, including one also serving as translator. FGDs and interviews were held in Bengali or in a mix of Bengali and English (all but 6 interviews). Interviews were audio-recorded apart from two where notes were taken instead. Audio files were transcribed and translated by experienced transcribers and translators. Data collection and initial data analysis happened iteratively followed by thematic data analysis [33]. An initial intermediate analysis of the FGDs allowed to follow-up and deepen emerging themes in subsequent interviews and also led to additional interviews with non-workshop participants. Transcripts and notes were coded in a qualitative data analysis software (NVIVO), then memos were written on different topics, discussed among the author team and collated into the themes presented below.

## Results

**Fig. 2:**
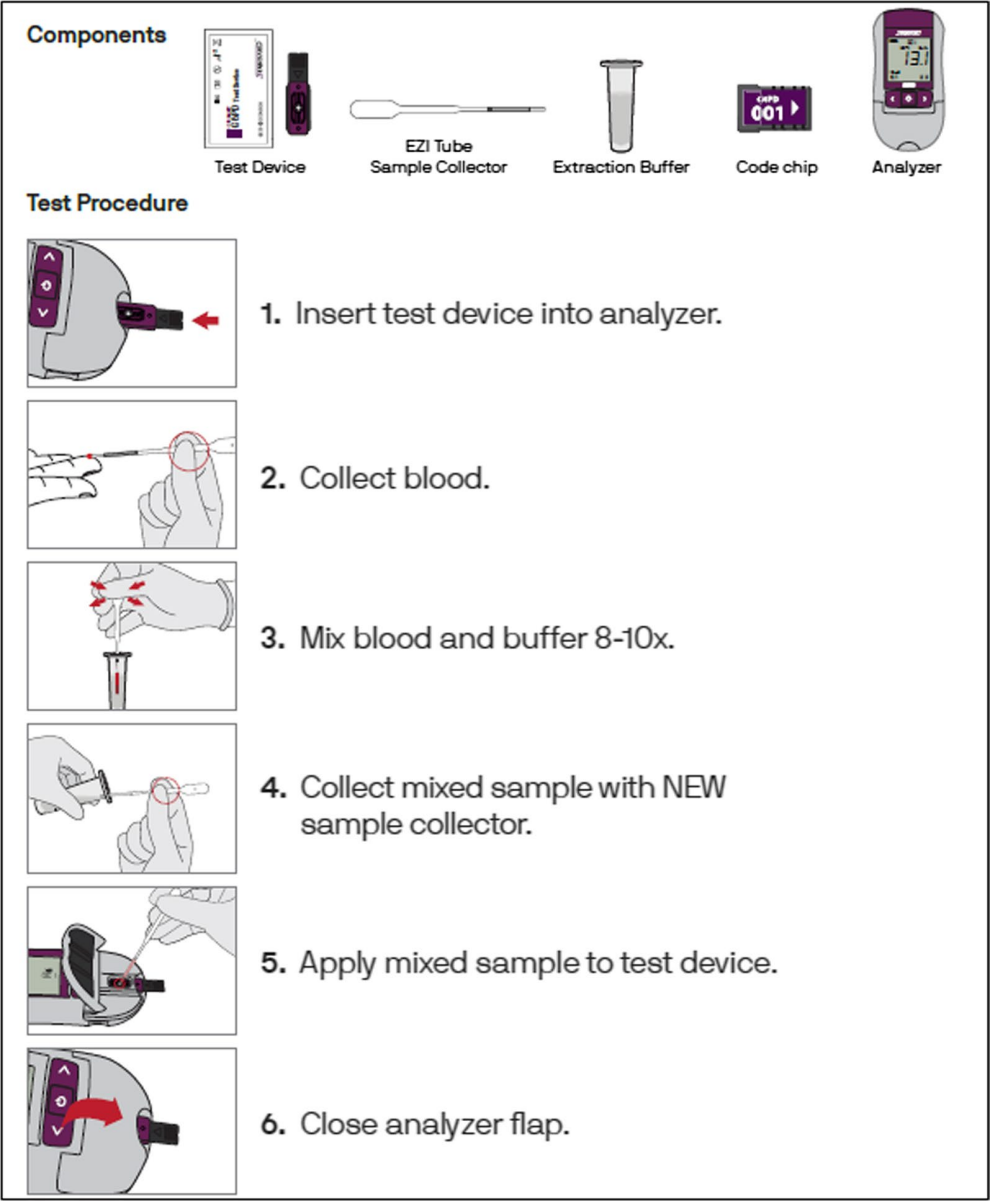
Schematic overview of G6PD testing using the STANDARD™ G6PD test (the biosensor) (reproduced with permission from SD Biosensor Diagnostics Inc., South Korea)

Study findings are grouped below by major themes. The first theme highlights what it would take to introduce the biosensor into routine laboratory and clinic practice, including technical aspects of the biosensor, supply chains and shelf life concerns, considerations over workload and training requirements and re-conceptualizing the risk associated with *P. vivax* in routine care. The second theme highlights how the context of elimination complicates diagnosis and introduction of new technologies, including how remoteness, challenging weather and a moving target require flexibility, how accessibility and meaning of diagnostics need to be considered, how there are simultaneously too many and too few patients to implement G6PD testing, and how to keep malaria visible whilst proving malaria is eliminable. To illustrate the technical aspects, a schematic outline of G6PD testing with the biosensor is shown in Fig. 2.

### Introducing the biosensor into routine laboratory and clinic practice

#### Technical aspects of the biosensor: ease of use, sample preparation and storage requirements

The LTs appreciated the usefulness of the biosensor results, its portability and ease of use (FGD1, 3). However, compared to existing portable diagnostics, such as the glucometer and malaria RDT, some technicians found the sample preparation procedure complicated. A laboratory technician outlined the requirement of additional steps, as follows:

*“for this we have to take blood then mix again then have to change the dropper and then again take the blood. It seems difficult to me. But for RDT, we put only buffer.*” (IDI3 LT DH)

The technician also identified that errors may potentially arise in busy clinics, such as inadvertently using the same sample transfer device twice (IDI 3 LT DH). In one of the FGDs, the LTs discussed concerns over using incorrect buffer volumes or insufficient mixing with blood hence eliminating the buffer step could improve the quality of the results (FGD3). Challenges of using the SD Ezi Tube sample collector (a sample transfer device developed by SD Biosensor) instead of the more familiar and more accurate micro-pipette were also discussed, since this would fit better with users who routinely collect venous blood rather than finger prick (IDI2 LT UHC, IDI6 LT UHC). Some workshop participants found the sample collector to be fiddly to use, for example presenting risks of aspirating air bubbles when using it in a hurry in the context of a busy clinic.

Concerns over electricity interruptions, battery replacements, humidity, storage conditions, maintaining cold chains and temperature (storage should be between 2–30 °C, but the test can be performed in the 15–40 °C range) especially, in remote locations where refrigerators or electricity are not always available or functioning also featured in the discussions (FGD1, FGD4, IDI7 nurse, IDI2 LT UHC, 3). They resulted in suggestions to allow for charging the device by direct connection to the main electricity (FGD1), and to provide a back-up device (FGD2, IDI15 LT UHC).

#### Supply chains and shelf life concerns

The LTs recounted experiences with poor quality and stock-outs of other testing supplies and expired drugs. This might explain concerns expressed regarding the 12-month shelf life of test strips and buffer of the biosensor (IDI6 LT UHC, IDI11 LT UHC, FGD2). During a FGD, a LT highlighted how the practical shelf life of a device in its intended end-user setting is shortened by the time taken during the supply and distribution process. This means that a stated 12-month shelf-life may only correspond to a six month shelf-life in a clinic:

*“It happens that a device arrives at Dhaka and remains there for a long time. It then takes time to reach the District level at which point it becomes a "short duration" device.”* (FGD2 participant)

According to the technicians, they are held accountable by their superiors for not using the device properly and are not able to provide timely care to patients. Technicians mentioned having been requested to overcome supply chain challenges, e.g. one technician was sent to the local market to buy missing items (IDI11 LT UHC), which would not be an option for the biosensor supplies. The participants of FGD2 recommended providing supplies prior to the malaria peak season (FGD2, IDI6 LT UHC) and to request accelerated shipping of supplies from the central level (FGD2).

#### Considerations over workload and training requirements

Various technicians expressed concerns that might arise from high clinic workloads, mentioning for example conducting around 100 tests daily (IDI11 LT UHC), being the only laboratory technician in a health centre, in some settings being only available twice a week, and having vacant posts not been filled for years (FGD2). Adding the biosensor to existing work therefore caused concerns, especially during the malaria season, and potentially causing delays in turn-around times and adding record keeping work (FGD1, 2, 3). Additional staff would be needed to provide results within 24 h (IDI11 LT UHC). Easy monitoring and minimum reporting efforts for the biosensor would be appreciated, as noted by a nurse at a district hospital (IDI1 nurse DH).

The workshop participants emphasized that it would be important to ensure sufficient training on the biosensor and the implications of G6PDd to staff across the health system, not just to LTs, but also to doctors (who prescribe and order tests), fieldworkers (who conduct RDTs and can motivate patients to seek testing), and nurses (who are available outside laboratory opening hours) (IDI11 LT UHC, IDI2 LT UHC, IDI3 LT DH, IDI5 doctor UHC; FGD3).

Laboratory technicians recommended conducting refresher trainings every six months or so, especially because patients with *P. vivax* malaria are few (IDI22 LT central laboratory, IDI16 LT UHC, IDI10 LT UHC).

#### Re-conceptualizing the risk associated with P. vivax in routine care

Currently, health workers at the community level refer patients with high *P. falciparum* parasite counts or severe symptoms to UHCs for treatment (IDI4 SACMO UHC, IDI20 programme officer, FGD3). Referral is challenged by cost, distance and time required and, especially during the rainy season, transportation infrastructure (FGD 2). If the biosensor were implemented at UHC levels but not at community level, health workers would have to refer *P. vivax* patients in the absence of severe symptoms or high parasite counts (IDI17 programme officer). *Plasmodium vivax* is not perceived as risky (IDI16 LT UHC). Since there is widespread experience with malaria symptoms (or the lack thereof), diagnosis and direct treatment at the community level, people might not be willing to travel for an additional test and treatment if symptoms are not severe, because it does not align with their long experience and knowledge of their malaria (FGD3). Additional communication around the likelihood of future *P. vivax* relapse and the risk of radical cure treatment for patients with G6PD would be important to encourage patients to pursue their care at the referral centers (IDI21 programme officer). This also means convincing patients to invest scarce time and resources for travelling and potentially facing transportation challenges, in the absence of severe symptoms (IDI17 programme officer).

### The context of elimination complicates diagnosis and the introduction of a new technology

#### Remoteness, challenging weather and a constantly moving target require flexibility in response

In Bangladesh, case investigations of all types of malaria cases is recommended, as per WHO guidelines [34]. A programme officer explained that coordination between staff at different levels of the health system is required and the response is complicated by lack of resources and staff (IDI17 programme officer). Programme officers are caught in a cat and mouse game, responding to hot-spots that keep moving from one region to another (IDI17 programme officer).

A programme officer outlined how donor funding is preplanned and not flexible enough to respond to such moving targets or changing needs. Government funding is more flexible but not necessarily available (IDI21 programme officer). According to the programme officer, 90% of malaria control in Bangladesh is donor funded and both the NMEP and local NGOs struggle with ensuring funding for diagnostics in the context of decreasing patient numbers. He explains how ensuring funding by the Global Fund for very low numbers of malaria patients requires creative ways of presenting disaggregated data (IDI21 programme officer). It might even lead to undesired consequences, such as overdiagnosing, to ensure funding continues (IDI15 LT UHD), and requires efficient use of limited resources to enable staff to travel to remote locations (IDI19 programme officer). Sometimes health care workers need other forms of (emotional) support or need to deviate from the WHO guidelines to make case investigation work:

*“if we have heavy rains, your staff cannot go [to do case investigation], so you cannot force them(..) otherwise, in those places, if I am being posted there, I will not do any work after one year. (…) So you have to think about their side. (..) the care provider needs also some sort of... backup support, emotional support. (…) Some places we can tell them '(…) you don't need to go there. Form a local team.' Try to solve the problems by themselves. (…) Sometimes they find a way. Even if not fully appropriate, but at least they find a way.” * (IDI19 programme officer)

#### Centralized or peripheral deployment of biosensor? Considering accessibility and meaning of diagnostics

According to the laboratory technicians, only a few patients are diagnosed with *P. vivax* and much of the malaria burden is concentrated in remote, border or conflict regions. This will complicate selecting the G6PD testing sites and managing the device, number of biosensors, expiry dates and packaging of the test strips which currently come in packs of 25 (FGD3, IDI10 LT UHC). Some UHCs diagnose about two *P. vivax* patients per month (IDI11 LT UHC) and others none:

*“…since vivax cases are relatively rare, and in some places more or less present, in some places it is absent. Where it is not found, the devices will expire. Twenty-five* [biosensor] *devices will be provided but not a single one used.” *(FGD3, participant 2)

During the FGDs, LTs discussed that currently, government and NGO health workers conduct malaria RDTs, diagnose and treat patients at the community level, hence most patients do not reach the sub-district/district level laboratories where the LTs work. However, FGD3 participants had concerns that patients usually diagnosed and treated at the community level might not be willing to travel for additional tests and treatment if G6PD testing and shorter course radical cure were centralized at UHC level (FGD3), mirroring similar concerns expressed in interviews (see above).

A programme officer (IDI21) illustrated that for different health workers, diagnostics can represent different things, namely ensuring an NGO’s financial survival, while at the same time overburden NGO workers with workload, or representing just one task among many others for governmental community level staff (CHCP):

*“…actually NGOs’ main focus is to detect these patients, and they have to show it - for getting their funds, so they are very focused on that. On the other hand, for the CHCP it is an added function because they have a lot of other activities. So they may not actually give due importance to diagnosing malaria. So maybe it is not working in the way that we actually thought*. (IDI21 programme officer)

According to programme officers, this has implications for who should be responsible for the biosensor diagnostic device (IDI17, 21). The NGO that prioritizes it as a matter of their survival but then might be overworked (IDI21 programme officer) or fudge numbers if patient numbers go down (IDI15 LT UHC)? Or the government facilities with many competing priorities but also existing expertise? According to a programme officer, “*ultimately, the community clinic [CHCP] should be the main center for management of malaria patients”* because these settings are more sustainable, even though they would require strengthening (IDI21 programme officer).

#### Too many and too few patients to implement G6PD testing

Participants emphasized the benefit to patients of testing for G6PDd and its necessity to reach elimination targets in 2030 (FGD3; IDI15 LT UHC; IDI22 LT central laboratory):

*“this* [testing biosensor] *is a new concept for our country. So, especially since primaquine has had that many, many contradictions with it, or that are involved in it, sometimes it is not known if there is severe anemia, even if someone dies. So it is a life-saving technology for us” *(FGD3 participant)

However, it was reported that there are simultaneously too many and too few patients to implement a test for G6PD like the biosensor. Too many patients as per FGD participants concerned about added workload (FGD1, 2, 3), and a former member of the Malaria Technical Committee in Dhaka recounting the discussion to not track every patient for G6PD testing during the creation of the national malaria guidelines in 2016 due to cost:

*“Bangladesh is not in that position now, to do G6PD for every patient. everywhere in Bangladesh. (..)... the case was so much... so high. (…). it is very costly also.* (IDI17 programme officer)

Malaria is almost entirely donor funded and so funding for the biosensor and related costs including reagents is a concern (IDI17 programme officer).

Conversely, programme officers mentioned various aspects for which there are too few *P. vivax* patients to acquire funding, justify staffing, storage space and the work required to create new policy with G6PD testing as a priority. They highlighted their challenges in keeping malaria visible and a priority in the face of declining patient numbers. A programme officer outlined that the significant reduction of positive tests resulted in the providers assuming that malaria was gone, and patients interpreting negative malaria RDT results as a failure to diagnose the disease, threatening the provider’s reputation and in turn resulting in less testing:

*“...earlier, they [healthworkers at NGOs] tested 10, found 7 positive. So 70% positive. But gradually it happens that they did 100 tests, and found 1 positive. So very low. From 70 to 1%. So two things happened: one is that the worker thought that there is no more malaria. On the other hand people who used to come there, when they were found negative and didn't get anything, they said 'why you are testing? You cannot identify the disease. You cannot diagnose the disease. Then why do you take the blood? I don't like it.' So both from the recipient end and the provider end they started to become reluctant [to test and be tested] and probably they were not testing.” *(IDI21 programme officer)

This dynamic led to an increased delay between the onset of symptoms and testing from 5 to 15 days at which point malaria-infected patients would have developed gametocytes that propel onwards transmission (IDI21 programme officer).

The programme officer (IDI21) argued that currently *P. vivax* and G6PDd are not given much priority since there are fewer patients with *P. vivax* than with *P. falciparum*, and it is a more benign form of malaria. There is little follow up on PQ treatment adherence (IDI17 programme officer), and the last therapeutic efficacy study on CQ and PQ in Bangladesh that the programme officer was aware of was conducted in 1977, so essentially policymakers do not know how effective CQ and PQ are (IDI17 programme officer) and more recent evidence is not mentioned [17]. As a result, little evidence is collected routinely on *P. vivax*, G6PD, adherence and possible complications of PQ treatment (IDI23 programme officer), despite the importance of such evidence informing changes to routine policy and practice:

*“… first challenge is that to collect information (...) evidence. To see what is the status of the vivax. Then you go for the status of G6PD.” * (IDI18 programme officer).

#### Keeping malaria visible whilst proving malaria is eliminable

According to a programme officer, work would have to be undertaken to ensure that the burden of *P. vivax* relapses remains visible and to explain the importance of *P. vivax* radical cure for malaria elimination, but that this would be essential to acquire donor funding, establish G6PD testing and radical cure treatment infrastructure, and uphold political will and leadership (IDI19).

Another programme officer illustrated the effort it took to change the status of malaria from controllable to eliminable and the national approach from focusing on endemic districts to hotspots. First, the programme officer had to make hotspots visible, i.e. produce data stratified by district, subdistrict, union and village to justify that elimination was possible and to generate funding (IDI21 programme officer). The programme officer developed interventions to prove that malaria could be eradicated; he mobilized health workers, involved communities and created partnerships with other communicable disease programmes, NGOs and military officers to prove the ‘eliminable’ character of malaria. Finally, he convinced local scientists and policy makers at the Ministry of Health and ultimately the Global Fund that Bangladesh should embark on the eliminating malaria.

According to the programme officer, the elimination approach also means that every malaria patient becomes a priority, whether *P. vivax* or *P. falciparum*, whether severe or not:

“*malaria is malaria*” (IDI21 programme officer).

## Discussion

This study aimed to improve the understanding of user perspectives on the feasibility of implementing the biosensor for G6PD testing in Bangladesh, in the event that the NMEP switches from the current 14 days PQ policy without G6PD testing to shorter course radical cure regimens with G6PD testing. While study participants emphasized the life-saving potential of the biosensor, they were concerned about available resources, in particular staff time and workload, limited follow-up capacity for ensuring PQ adherence, documentation burden, supply chains and the impact of supply delays on shelf-life. They were also concerned about technical aspects of the device, including the sample collector, battery life and the devices storage conditions if kept at the community level.

Qualitative research shows that implementing diagnostics at the point of care is not straightforward, particularly in economically constrained health systems. It requires strong and well-funded health infrastructure and systems [27], as well as providers who are undergoing continuous professional development and are actively engaged in the policy process [35]. Such requirements were indeed raised by this study, both at the programme officer level (e.g. efforts required to raise funds and the need to make careful decisions about how and where to implement the biosensor) and at the LT level (e.g. training requirements, refresher trainings every season, etc.). Further, improvisations by health workers at every level of the healthcare system play a central and often structural role in fragmented health systems to cope with uncertainty, such as stock-outs, and need to be considered when implementing new technologies [36]. The study participants pointed out the need to organize well the entire supply chain of biosensor devices, with proper management of quantities and timely deliveries. They also noted the need to engage a wide variety of healthcare professionals in training on the biosensor beyond those conducting it. Implicitly, this acknowledges the importance of functioning relationships between technicians and doctors or nurses, between providers for referral, and effective collaboration to ensure diagnostic processes at the point of care [28].

Additionally, the study shows how a context of elimination and a disappearing disease add to these dynamics and perspectives in four important ways. First, the remoteness of many malaria endemic sites, as well as changing malaria hotspots require flexibility and improvisation by policymakers and health workers. Eliminating malaria is aiming at a constantly moving target. This heterogeneity makes it more difficult to introduce any new diagnostic including G6PD testing, and to budget for related supplies, human resources, or decide on deployment. As others have argued before, malaria control must be managed locally, in everyday practices [37] and cannot be achieved through commodities alone [38].

Second, declining patient numbers in elimination settings raise issues regarding healthcare providers having a low index of suspicion of malaria. These data show that as the numbers of malaria patients decline [32], malaria testing can fall from the practice of both health workers and patients. Concurrently communities continue to be exposed to parasites, and elimination becomes difficult. Ensuring that malaria diagnostics are integrated into a package of diagnostic services for other febrile illnesses, so that a negative malaria test result guides further diagnostic steps rather than being a final diagnostic conclusion [39], would increase the relevance of testing to community members given the low incidence of malaria. In the case of human African trypanosomiasis (HAT), anthropologists found that a diagnostic reflex among healthcare workers is stronger when they are in the habit of testing and have time and space to consider symptoms and that this reflex also exists among communities and patients. A diagnostic reflex can be trained in times of elimination by treating detection of events as important learning opportunities and ensuring multidimensional access to diagnostics across the health system [40]. These data show that in order to make new technologies such as the biosensor accessible, malaria needs to be made and kept visible. The results of this study point to different strategies towards achieving this, such as: demonstrating that malaria can be eliminated, mobilizing political will and different actors (NGOs, government clinics, border control guards, police, tribal community leaders, Ministry of Health department), for instance, to find out why a potential resurgence exists and what is happening to testing practices, creating partnerships, generating research-based evidence and communicating on the risks and benefits of PQ treatment and severe reactions to PQ.

Third, the biosensor requires new forms of evidence to justify its introduction into policy. The study revealed current perceptions that there are simultaneously too few and too many *P. vivax* patients to implement G6PD testing owing to challenges of funding, workload and complex testing infrastructure. Efficient communication about the need to eliminate and properly treat *P. vivax*, radical cure treatment, and G6PDd is vital to ensure that the relevance of the biosensor is appreciated and its value recognized. Generating such evidence and initiating these discussions therefore becomes an important part in the decision-making process of implementing the biosensor and shorter course radical cure treatment to support *P. vivax* elimination. It would also fuel ongoing debates about priorities at policy and practice levels (“*P. vivax* is benign”, versus “malaria is malaria” and “all forms are equally relevant”; “radical cure is a societal not an individual concern”), which were reported in this study and which persist in Bangladesh without consensus [41].

Fourth, declining patient numbers combined with the relative complexity and cost of the test –in comparison with simple malaria RDTs for example- complicate the process of deployment. Should G6PD testing and radical cure with shorter course regimens be centralized with qualified LTs and doctors at UHC and DH level, or rather be done at community level and in remote areas, closer to the malaria patients? The study findings provide various considerations to answer this question and highlight barriers that would need to be addressed. One important aspect is the difficulty in referring patients to the UHC or DH level in the absence of any severe symptoms and to manage the health staff’s workload if the biosensor was implemented at that level. In areas pushing for malaria elimination, additional resources towards reinforced surveillance, for instance during rainy seasons, may be required to support and complement the ongoing efforts of the staff overseeing routine care. More generally, the results show that any decisions about deployment must take into account the technical aspects, including throughput, packaging and shelf life, of the device as well as infrastructure, epidemiological, behavioural and ecological factors of the settings.

Questions around deployment and implementation strategies need to address the fact that diagnostics have different meanings for different actors (e.g. at programme level) and users (e.g. in clinics, laboratories and at the POC). The study results indicate that implementing and using the biosensor can mean offering better care and facilitating elimination of *P. vivax* malaria, while strengthening one’s expertise and professional role in the healthcare system, it can help to mobilize support/funding for an NGO, it can be perceived as an indicator of good quality care, but also as a tool that increases workload in already strained work environments. Research on malaria RDTs, for example, shows the importance that such meanings and values take on for adhering to test results, and how local knowledge and understanding of good clinical practice interact with the availability of drugs and contexts of scarcity in determining the meaning of these diagnostics for health workers and patients [36, 42–44]. For the policymaker, the biosensor signifies extra work obtaining funding and setting up implementation plans, guidelines and workflows. For the health worker, the biosensor may add workload too but also allows a safer prescription of shorter course PQ or TQ.

### Study limitations

This study focused on LTs and a small number of programme officers and decision-makers around training workshops. Future research could assess how user expectations and concerns that were highlighted compare with reality after a few months of using the assay. Future research should include community level providers, NGOs as well as patients which were not included in this study. Without doubt, similar studies involving healthcare staff at other levels (i.e. community health workers, doctors, nurses, NGOs) as well as patients would complement the findings of this study to support the design of efficient implementation strategies for G6PD testing and new radical cure approaches, and to improve care of *P. vivax* patients and move towards elimination of this malaria parasite species.

## Conclusion

This study highlights how qualitative research methodologies can reveal important factors to consider when implementing a new diagnostic test (along with improved treatment regimens in this case). Concerns raised regarding the technical and logistical issues surrounding the test should be addressed by future product designs, adequate training and strengthening or streamlining supply chains. Issues regarding difficult patient referral may require a reconceptualization of risk of *P. vivax* treatment combined with good communication and advocacy, and LT opinions point to the need of training all involved health professionals which should obviously be followed by careful planning of staff interactions at all levels of the health system. To trigger policy change, it will be critical to solve ongoing debates regarding priorities at policy and practice level and advocate for means to efficiently and safely implement *P. vivax* radical cure. To judge the value of G6PD diagnostics, evidence-gathering at the end-user level will be needed to ensure that *P. vivax*, PQ treatment, and G6PDd remain visible.

## Supplementary Information

**Additional file 1**: FGD discussion guides.

**Additional file 2**: Interview guides health workers.

**Additional file 3**: Interview guides programme officers and decision-makers.

## Data Availability

The datasets used and/or analysed during the current study are available from the corresponding author on reasonable request and only with the necessary editing to ensure anonymity of participants.

## Abbreviations

BRAC: Building Resources across Communities
CBC: Complete blood count
CDC: Communicable Disease Control
CHCP: Governmental community level staff
CQ: Chloroquine
DH: District hospital
FGD: Focus group discussion
FIND: Foundation for Innovative New Diagnostics
G6PD: Glucose-6-phosphate dehydrogenase
G6PDd: G6PD deficiency
GFATM: Global Fund to Fight AIDS, Tuberculosis and Malaria
icddr,b: International Centre for Diarrhoeal Disease Research, Bangladesh
IEDCR: Institute of Epidemiology, Disease Control and Research
LT: Laboratory technician
NMEP: National Malaria Elimination Program
POC: Point of care
PQ: Primaquine
RDT: Rapid diagnostic test
SACMO: Sub-assistant community medical officers
TQ: Tafenoquine
UHC: Upazila Health Complexes
WHO: World Health Organization
