# Supplementary material for: Implementing radical cure diagnostics for malaria: user perspectives on G6PD testing in Bangladesh
Source: Malar J. 2021 May 12;20:217. doi: 10.1186/s12936-021-03743-w (PMC8114691; doi:10.1186/s12936-021-03743-w)
Supplement: Supplementary file 1 — Additional file 1: FGD discussion guides. [file 12936_2021_3743_MOESM1_ESM.pdf]

Disclaimer: Developed by University of Maastricht (the Netherlands, Nora Engel & Cristian Ghergu) in collaboration with FIND (Switzerland), the Menzies School of Health Research (Australia) and the icddr.b (Bangladesh), for the use in training workshops on the STANDARD™ G6PD test. This document may be freely reviewed, quoted, reproduced or translated, in part or in full, provided the source is acknowledged.

Focus Group Discussion Guide

1. Objectives and logistics

Objectives:

The objective of the FGDs is to gain insights from participants about the experience with, usability of, and opinions about potential implementation of the G6PD Biosensor in clinical environments and the malaria care continuum in Bangladesh.

- To understand the needs and preferences of participants regarding the (1) use and (2) implementation of the G6PD biosensor.
- To explore how the G6PD Biosensor may be aligned with their needs and work environments.
- To explore ideas about possible challenges and facilitators to implementing the test in clinical settings.

Logistics at FGD site:

- Informed consent has to be taken.
- Materials: Name cards, ID tags, writing supplies for observer and participants, envelopes for informed consent forms, enrolment log (to record names and IDs), post-its, refreshments for participants. Have at least one biosensor at hand, so participants can use as a prop if they want to discuss specific aspects of its use. Have training materials at hand for the same purpose.

2. Instructions of how to organise this discussion (for the moderator):

Introduction:

- Purpose of session; general outline of session:

  'Thank you for being here. The purpose of this session is to discuss your experiences with the biosensor, and how it could be implemented in your work environment and routine. First you will discuss some of the things you liked about the training and biosensor. After that you will be encouraged to discuss some of the potential challenges of using the biosensor and implementing it in your daily work routine.'

- Recording and confidentiality:

  'With your accord, the session will be recorded for future reference. We will not identify you by your name in our report, and all information recorded is considered confidential. The records will be kept at a safe place. '

- Oral consent:

  'Would it be okay to record this session?'

- Ground rules of group discussion:
  'We would like to encourage everyone to participate in the discussion. There are no wrong and right answers: the study is explorative and not intended as a clinical audit or assessment; Please allow for one person speaking at a time; we ask that you turn off, or silent, your mobile phones. If you must respond to a call, please do so as quietly as possible and rejoin us as soon as you can.'
- Participants introduce themselves (if necessary use of name cards) name, profession/ affiliation.
  'let us have a quick round of introductions.'

Post it exercise guidelines:

- Explain the purpose, and exercise

  'To help guide the discussion, and ensure everyone has their chance to contribute, we will do a post-it exercise. For the exercise, we will distribute to everyone a couple of post it notes and a marker. I will then ask you a question about the workshop and biosensor and you will have 5 minutes to write up your answer. You are encouraged to write multiple answers, each written on a separate post-it. After 5 minutes, I will collect them and arrange them on this whiteboard. Then, we will discuss your answers in more detail. We will do this exercise two times over the duration of the session.'

- Distribute the post-it notes.
- Ask question a, repeat that they can write multiple answers, one per post-it.
- Short silence in which participants write down ideas. After 5 minutes collect the answers and arrange them according to emerging themes.
- Clarify any unclear notes/words.
- Moderate discussion based on emerged themes and ask probing questions.
- Make a summary of the main results.
- Ask if anyone has comments or anything to add.
- Ask question b. Repeat process above.
- Ask question c.
- At the end, provide a summary of the discussion. Ask if any of the participants have other comments, or anything to add.
- Debriefing:

  'Any last comments/questions/recommendations about the FGD and study?'

3. Questions

a. *'What are the things you liked about the test, or found to be useful?'*

b. *'What are the biggest challenges that you can think of when using the G6PD test?* (subquestions for each theme individually)

   - *Please explain more.* (if relevant: *example?)*
   - *What could be solutions to address this?*
     - *By training?*
     - *By test design?*
     - *Other (institutional changes?)*

- *How feasible are these solutions?*

*Follow-up question (with probes for aspects left unaddressed so far):

c. *What are the biggest challenges that you can think of when the G6PD is now newly introduced for use in your daily work in your health complex? (i.e. challenges you would see for the process of training staff, delivering the new materials, making everything ready for starting its use?)*

    - *Challenges with having to train all staff?*
    - *Challenges with how to assign/modify the responsibilities of performing the test (who, etc.)?*
    - *Challenges with explaining well (by the trainers) and understanding well (by the HWs) how this will change the patient treatment decisions?*
    - *Challenges with having to change the treatment guidelines and communicate the new version?*
    - *Others?*

4. Topics under which answers can be grouped for discussion (flexible, guiding)

For the use of the biosensor:

Design of biosensor and usability	Timing	Patients' experience	Interpreting and using results at POC
Additional materials required	Cost	Workload, changes in responsibility of performing the test	Infrastructure, supply chain
Quality control	Training	Infection risk	Reporting, administrative or operational aspects

For the introduction/implementation of the biosensor:

Having to train multiple staff across the country	Having to revise and communicate modified testing and treatment guidelines	Having to modify roles and responsibilities of lab and clinical staff	Having to explain well (on trainers side) and understand well (on HWs side) how to use test results
