# Supplementary material for: Implementing radical cure diagnostics for malaria: user perspectives on G6PD testing in Bangladesh
Source: Malar J. 2021 May 12;20:217. doi: 10.1186/s12936-021-03743-w (PMC8114691; doi:10.1186/s12936-021-03743-w)
Supplement: Supplementary file 2 — Additional file 2: Interview guides health workers. [file 12936_2021_3743_MOESM2_ESM.pdf]

Disclaimer: Developed by University of Maastricht (the Netherlands, Nora Engel & Cristian Ghergu) in collaboration with FIND (Switzerland), the Menzies School of Health Research (Australia) and the icddr.b (Bangladesh), for the use in training workshops on the STANDARD™ G6PD test. This document may be freely reviewed, quoted, reproduced or translated, in part or in full, provided the source is acknowledged.

Interiew guide healthworkers

<table>
<tr><th colspan="2">Questions/Probes</th><th>Information we are looking for</th></tr>
<tr><td colspan="2"><u>How does a regular day of work look like for you?</u>
o Chronological account. If fitting, probe for responsibilities, interactions.</td><td>Roles
Responsibilities
Interactions (staff, patients, diseases)</td></tr>
<tr><td rowspan="3"><u>How would you describe the process of diagnosing and treating malaria?</u></td><td>[Screening]
o From the moment a patient enters the (institution) with malarial symptoms, what happens? Can you please go through steps?
- What do you discuss? (What questions do you ask? Do patients also talk about other things?)
- What do you record?
- Who is involved?
How do you decide if the patient needs a diagnostic?/ are all patients presenting with fever tested?</td><td rowspan="3">Experiences and challenges of seeking care, screening, testing, diagnosing, treating malaria in work context.

• Steps performed
• Relevant actors involved, and their interaction: patients, laboratory staff, tests/other technology involved, referral mechanisms, others

• Experience and challenges with diagnostic process generally, and tests in particular

• Information coming up at different steps, recording, decision making

• Possible improvements

• Primaquine treatment practices; understanding of risks associated with the treatment; patient responses

• Interactions with patients, communication about diagnostics, results, risks, etc</td></tr>
<tr><td>[Diagnosing]
o What tests do you use for diagnosing malaria?
o Who is involved? (Lab?)
o Time required? (TAT, until you get results)
o How do you record?
o Does it ever happen that patients demand a specific test? When? Who? Why?
o Examples? Common/unusual decisions?</td></tr>
<tr><td>[Treatment decisions?]
o Once you have the test result, what happens next?
o Who is involved?
o How do you treat for Pf and Pv and mixed?
- When are decisions made?
- Who does them?
- Based on what information?
o Does it ever happen that patients demand a specific treatment? When? Who? Why?
o Examples? Common/unusual decisions?
o Does it ever happen to initiate treatment without testing?
[Primaquine treatment]
o Do you use Primaquine treatment on a regular basis?
- Since when?
- How do you decide to use it? On what patients, when? Other considerations?)
- Who gives it? What information accompanies it?
- What are your experiences with the treatment? How about challenges?
- What do patients think about receiving primaquine treatment?
- Are there any challenges with the costs?</td></tr>
</table>

	- Do you think it is a useful treatment?<br>- Did you ever switch patients<br>o To another treatment midway?<br>o Abort treatment<br>o Why/how?<br>o Is G6PD deficiency or haemolysis a concern when deciding on treatment? How do you include it in your treatment decision?<br>o Can you let me know the key symptoms suggestive of an acute haemolytic episode?<br>- Did you ever experience or hear about patients that had drug induced haemolysis after getting treated with primaquine?<br>- How are haemolysis risks addressed? Examples (testing? discussions with patients? concerns)?<br>- Who is involved in monitoring (treatment follow-up)? How is it done? (during/after treatment, what do patients do, where do they go, who else is involved?). What happens if a patient has haemolytic symptoms?<br>o<br><br>Implementation processes<br>• Steps performed (trainings, resources, information, support, accountability mechanisms etc)<br>• Actors involved<br>• Narratives about change (preferences, considerations for, or against)<br>• Decision making process (formal and informal)<br>• Adaptations made during implementation, and after	
Since you work here, have there been any changes in the diagnostic and treatment process in the past?<br>o What kind? (examples: tests, protocols, treatments)<br>o Why?<br>o How are you informed about changes? Can you please go through the steps of the change? (trainings, resources, information, support, etc)<br>- Who was involved? How?<br>- How did it change your day-to-day practice? Was there an opportunity to give feedback to the decision makers? Did you also have to change things in reporting or recording information? Examples?<br>o Do you have suggestions for improving future implementation of new guidelines/tests or treatments?		Let us start with the training?<br>o How did you find it?<br>- Training materials,<br>- Experience with trainer,<br>- Theoretical and practical sessions<br>- Duration?<br>- Anything else that stood out from the training?<br>o Usefulness? Preferences? Dislikes? (on delivery and content)<br>o Possible improvements?
What did you think about the workshop?	- What did you think of the biosensor?<br>- Would you consider the test useful? Why (not)?<br>- Who should use the test in your UHC? Anyone else? Why?<br>- What about other	STANDARD^{TM} G6PD test<br>• User perspectives on experience with G6PD Biosensor and training materials.

	- Would you use it? Or would you think it should be used by other staff in your facility, or staff at other levels of the health system? Which ones?<br>- Additional materials required?<br>- Preparing the device<br>- Blood collection, preparation, operation<br>- Displaying the results and interpreting them?<br>- Disposal of used consumables – challenge?<br>o Challenges?<br>Possible areas for improvement?	• Information on how would the STANDARD^{TM} G6PD test fit in the work environment and malaria continuum of care.<br>• Preferences and challenges regarding the new test: technology design and implementation.
	What changes would the G6PD biosensor bring into the malaria diagnostic and treatment process?<br>o What changes would the test bring in your current work environment?<br>o What changes would it bring in diagnosing and treating malaria? / And in using Primaquine?<br>o How do you think it would affect:<br>- linkage to care<br>- cutting/increasing diagnostic delay<br>- cost concerns<br>- accessibility<br>- (what would the patient think? - patient's experience of care<br>o How do you think the test should be best introduced?<br>- What changes would have to be made to the test, or training materials to make sure it is introduced and maintained successfully?<br>- How about changes in the hospital?/work environment?<br>- When introducing the test, who should be involved from the hospital? And in what way?<br>- What kind of training would you like to see done? (e.g. how long, contents of the training?)<br>- What kind of follow-up/supervision/refresher activities would you like to see done after the training?<br>o Would other things be needed, e.g. reduce workload for other activities, need of more staff, assign activities differently to the different staff? Etc.	
	- Final points	
