# Supplementary material for: Implementing radical cure diagnostics for malaria: user perspectives on G6PD testing in Bangladesh
Source: Malar J. 2021 May 12;20:217. doi: 10.1186/s12936-021-03743-w (PMC8114691; doi:10.1186/s12936-021-03743-w)
Supplement: Supplementary file 3 — Additional file 3: Interview guides programme officers and decision-makers. [file 12936_2021_3743_MOESM3_ESM.pdf]

Disclaimer: Developed by University of Maastricht (the Netherlands, Nora Engel & Cristian Ghergu) in collaboration with FIND (Switzerland), the Menzies School of Health Research (Australia) and the icddr.b (Bangladesh), for the use in training workshops on the STANDARD™ G6PD test. This document may be freely reviewed, quoted, reproduced or translated, in part or in full, provided the source is acknowledged.

Interview guide Programme officers

**Main questions, and probing questions**	**Information sought after**
▪ Could you please tell me about your job, and what you do on a daily basis?<br>o How about in the past?	<u>Introduction, localizing the respondent.</u><br><br>Role (present past); Involvement in malaria. – establish connection to either: development of guidelines (such as the Malaria treatment regimen), or implementation, or organizational capacity.
▪ Can you take me through the diagnosis and management chart for Pv?<br>▪ Implementation?<br>▪ Challenges?<br>▪ How was it decided to arrive at it? –any other aspects/alternatives considered to include?/changes from previous chart? (why?/why not include?)(probe for examples, tests?)<br>o The chart mentions that G6PD deficient patients are at high risk of developing haemolysis:<br>▪ How is that being deal with now in hospitals?<br>▪ Challenges? (possibilities for improvement?)<br>▪ How do you monitor/get to know about these?	▪ Pv and G6PD considerations, recommendations, barriers.<br>▪ Alignment between biosensor and needs, priorities as perceived by policy/protocols makers<br>▪ Process of creating and implementing the guideline for Pv.
**<u>Questions about the use of the G6PD biosensor in Bangladesh:</u>**<br><br>▪ Can you tell me what you know about the G6PD biosensor?<br>▪ From your experience, was the idea of implementing a G6PD test in Bangladesh considered before?<br>▪ If yes – can you tell me more about it? (how was the decision arrived at?)<br>▪ Considering the current situation in Bangladesh, do you think the biosensor should be implemented? (why not?)<br>▪ How do you think the test sh/could be implemented?<br>o At which level of the health system?	G6PD biosensor: considerations for implementation, process (including barriers) to implementation, recommendations.

o What type of staff should use it?<br>o What barriers to optimal use do you see?<br>o How would you ensure that the test is used correctly? (information delivery, training, guidelines, reporting, other) – probe further for each (who, what, why, barriers?)<br>▪ What do you think about the cost of adding this test in the test & treat algorithm, versus its benefits of avoiding risks of hemolysis in G6PD deficient patients?<br>▪ What other options are there for testing G6PDd, besides the biosensor? (qualitative tests for example)<br>▪ How would they compare with the biosensor?<br>▪ What would be your opinion about using one or the other in Bangladesh?<br><br>**<u>Questions about how best to introduce and implement the G6PD Biosensor in the country:</u>**<br><br>▪ For implementing this test in Bangladesh, what would be required to do?<br>▪ Any preliminary steps before large scale implementation?<br>o What would be needed at international/global level?<br>▪ Information, guidelines, studies, other?<br>o How about national?<br>▪ Specific steps required: e.g. need to convene stakeholders first, establish guidelines, training tools, etc. (discuss here what the process was for implementing any new tests in the past, revise the malaria treatment guidelines in the past, etc.)	
If time allows it:<br><br>o Could you tell us more about past experiences with the implementation of other <u>tests for malaria</u>?/other treatment regimens (eg RDTs)<br>▪ How were they implemented? (who was involved, considerations for implementation, evidence, implementation methods)<br>▪ Challenges?<br>▪ Recommendations?	Implementation process, narratives, for other tests.
Ending questions	Things we might have missed, other things to investigate, snowball?
